# Local Interpretable Model-Agnostic Explanations for Classification of Lymph Node Metastases

**DOI:** 10.3390/s19132969

**Published:** 2019-07-05

**Authors:** Iam Palatnik de Sousa, Marley Maria Bernardes Rebuzzi Vellasco, Eduardo Costa da Silva

**Affiliations:** Department of Electrical Engineering, Pontifical Catholic University of Rio de Janeiro, Rio de Janeiro 22451-900, Brazil

**Keywords:** explainable AI, deep learning, medical data, lymph node metastases

## Abstract

An application of explainable artificial intelligence on medical data is presented. There is an increasing demand in machine learning literature for such explainable models in health-related applications. This work aims to generate explanations on how a Convolutional Neural Network (CNN) detects tumor tissue in patches extracted from histology whole slide images. This is achieved using the “locally-interpretable model-agnostic explanations” methodology. Two publicly-available convolutional neural networks trained on the Patch Camelyon Benchmark are analyzed. Three common segmentation algorithms are compared for superpixel generation, and a fourth simpler parameter-free segmentation algorithm is proposed. The main characteristics of the explanations are discussed, as well as the key patterns identified in true positive predictions. The results are compared to medical annotations and literature and suggest that the CNN predictions follow at least some aspects of human expert knowledge.

## 1. Introduction

Artificial Intelligence (AI) and Machine Learning (ML) have become increasingly ubiquitous in the study of data from the medical domain. One notable example is the use of various Convolutional Neural Network (CNN) architectures and other Deep Learning (DL) models for tasks related to medical image classification and segmentation [1,2,3,4]. In particular, the use of such networks to detect metastases in patches extracted from Whole Slide histopathology Images (WSI) leads to performances that often surpass human pathologists [4,5].

The importance of such results is increased by the fact that pathology experts performing the same task can disagree in more than 20% of the cases [6]. This task is also described in the literature as tedious and time consuming, which makes it more prone to human error. The existence of ML/DL systems that can perform the task at very high levels of accuracy seems like a way to overcome this problem.

Since the demand for such pathologists is extremely high around the world, these AI systems could greatly aid in balancing their work load and allow for a more accurate and consistent diagnosis.

However, in spite of these promising results, there is in general no clear indication of what makes a particular ML system, such as a CNN, output a certain prediction or classification for a given patch. Several authors cite this black-box behavior as a central problem to be addressed in ML research concerning medical data, before AI can truly be adopted for this type of task in the medical world [3,7,8,9]. This issue, however, extends to many other areas where AI-aided decisions are critical and cannot be blindly trusted.

This scenario resulted in a growing number of publications regarding the development of techniques to explain decisions by AI models. This area of research, often termed Explainable-AI (XAI), has been blooming most notably since 2016, with both original papers and systematic reviews being recently published [8].

For image classification problems, specifically, several XAI techniques have been developed using different ideas. In many cases, the techniques involve backpropagation of the output to the input neurons using specific functions that generate the explanations or other specific operations conceived specifically for CNNs, as summarized by Alber et al. [10]. The explanations generated in these cases are heat maps where each pixel in the image receives a value according to how relevant it is for a given classification. Ribeiro et al. [9] developed a different approach of Locally-Interpretable Model-agnostic Explanations (LIME) where, in the case of image explanations, a given image is segmented algorithmically into superpixels, and the relevance of each superpixel for a given classification is determined using a linear model. The algorithm is model-agnostic as it only requires the outputs of the classifier for different images. In fact, LIME can be used for any image classifying system, not just neural networks, as it does not employ any specific backpropagation procedures or any steps specific to any individual model type.

Implementations of these techniques have become available recently as specific programming libraries were developed [9,10]. Although the need for XAI studies in medical imaging is clearly stated in the literature, there is still a lack of publications concerning experiments in this subject.

The main contribution of the present work is precisely to address this issue by applying an explainable AI technique to the problem of metastases classification in Whole Slide Image (WSI) patches, to analyze how a typically high-performing DL classifier makes its predictions and which parts of the image affect its decisions.

Among the many available techniques previously discussed, LIME stands out as a candidate for this study. Due to its model-agnostic nature, results of future studies, regardless of the models used then, could be more easily compared to the results presented here, which would not be necessarily true with the gradient and saliency-based techniques described in [10]. Furthermore, saliency heat maps have been shown to be unreliable explanations under certain conditions [11], which has not been cited as an issue with LIME.

Additional contributions here described involve the comparison of commonly-used image segmentation algorithms and their influences on the generation of LIME explanations for tumor classifications. A simpler image segmentation strategy involving square grids, devoid of any parameter fine tuning, is also proposed and tested.

The dataset related to this task is the Patch Camelyon (P-CAM) benchmark recently released by Veeling et al. [4], described in more detail in the next section.

## 2. Materials and Methods

This section describes the application of the LIME method, as well as the CNN models used and the dataset with images from WSI patches.

### 2.1. Dataset

Patch Camelyon (P-CAM) is a dataset developed by Veeling et al. [4]. It was derived from the Camelyon16 hematoxylin and eosin-stained WSIs. The original whole slide images were acquired and digitized with a 40× objective (corresponding to a pixel resolution of 0.243 microns) [6], and undersampled at 10× to increase the field of view for P-CAM.

**Figure 1:**
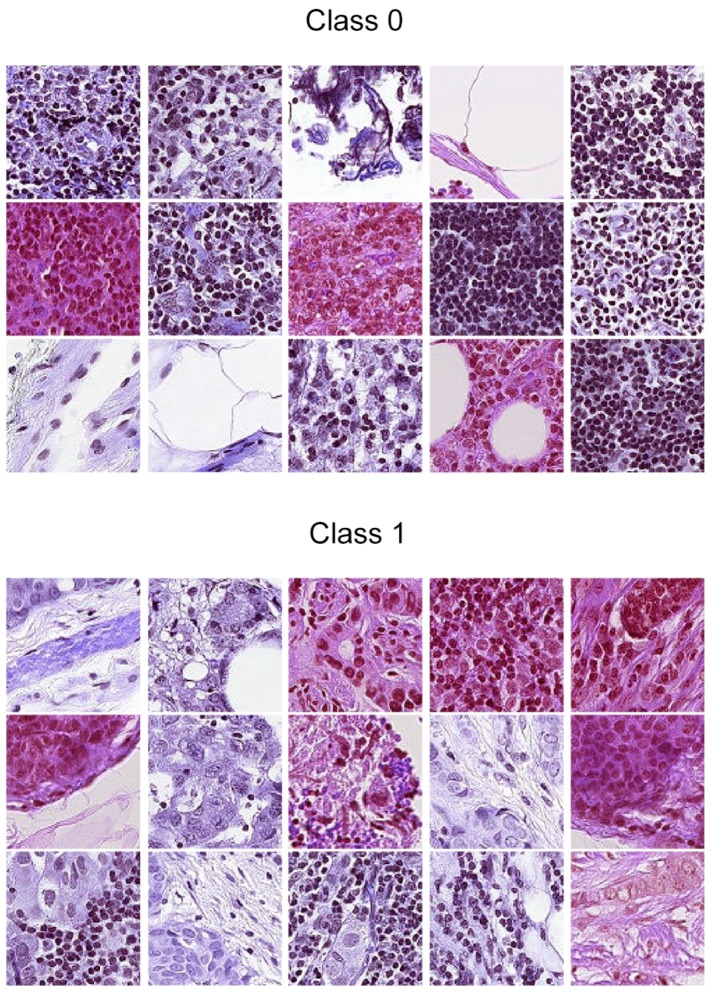
Samples from the Patch Camelyon (P-CAM) dataset by Veeling et al. [4]. Class 1 indicates that there is at least one pixel of tumor tissue in the center of the image (the center is a 32 by 32 pixel square in the middle of the patch), while Class 0 indicates the opposite.

The 96 by 96 pixel patches were extracted by Veeling et al. from the gigapixel WSIs by converting the slides to hue-saturation-value format (HSV), followed by blurring and filtering out patches that had saturation lines below 0.07, which was shown to exclude irrelevant background patches. The binary labels of each image corresponded to the presence or absence of at least one pixel of tumor tissue in the central 32 by 32 pixel square of each patch (0 meaning absence, 1 meaning presence). Tumor tissue outside this square did not influence the label. However, this outer region was maintained in the image, both to it possibly giving relevant context for classification algorithms and to enable the usage of certain types of fully-convolutional models that do not use zero-padding [12]. Class balancing close to a 50/50 divide was achieved by a stochastic hard negative mining scheme. The dataset was presented in two versions. The first one was a GitHub repository [12] containing the full dataset of 327,680 patches divided into training validation and test sets following the same division of the original Camelyon16 data. The second version was the same as the first, but removing duplicate images present in the original P-CAMdue to the probabilistic sampling of patches. This version was presented as a dataset for a Kaggle competition [13]. Besides the removal of duplicates, there were no differences between the splits or other aspects of the data. There were 220,026 labeled images in total for this version. For the remainder of this manuscript, whenever the P-CAM dataset or patches are mentioned, they refer to this second version. Figure 1 shows an example of some images for the dataset.

#### Medical Annotation

The original Camelyon16dataset also contained manual annotations of which parts of the image corresponded to the metastases. This annotation/segmentation was done and verified by students and expert pathologists at two different hospitals in the Netherlands (Radboud University Medical Center and University Medical Center Utrecht) [6]. These annotations were later on mapped to the corresponding patches by Veeling et al. and made available on the PCAM GitHub repository [12]. Throughout this manuscript, the medical annotations are referred to as “Seg” (Segmentation) in the figures and are visualized as a green transparent overlay placed over the patches or explanation figures. Green pixels indicate metastases, and white pixels indicate normal tissue. This can be seen, for instance, in Figures 6 and 7.

### 2.2. LIME

The proposed methodology makes use of the original LIME implementation by Ribeiro et al. [9]. This technique creates explanations by first dividing a given image into superpixels. These were clusters of pixels with similar colors, textures, or other characteristics, that gave local contextual information about some part of the image. A distribution of perturbed images was then generated by randomly hiding some superpixels.

The effect of image perturbations on the correct class prediction probabilities was then computed. Next, a linear model was trained with these perturbed data. The relevance of each superpixel for a given classification was then provided as weight values: positive values indicated the influence towards a correct classification, negative values the opposite.

**Figure 2:**
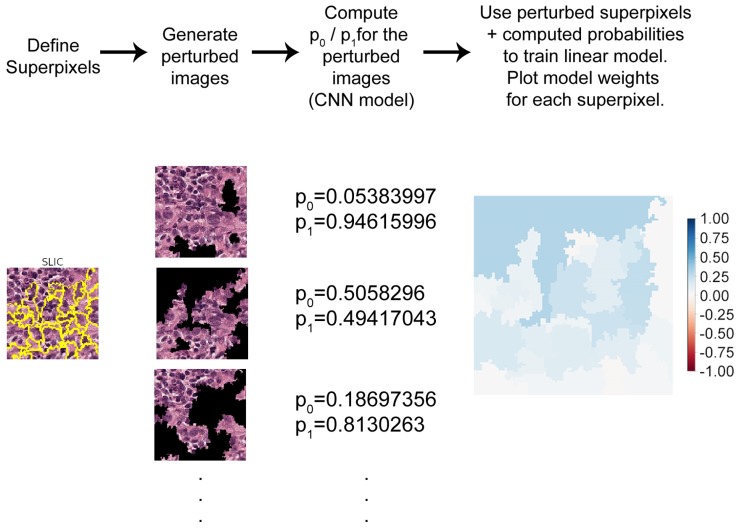
Diagram of the Locally-Interpretable Model-agnostic Explanations (LIME) algorithm in four steps. To generate explanations for a classification, the given image was first divided into superpixels. A distribution of perturbed images was generated and passed through the original prediction model to compute the classification probabilities. These probabilities and perturbed images were presented to a regression model that estimated the positive or negative contribution of each superpixel to the classification. The regression weights were then plotted in a blue-red color map.

An example of this procedure can be seen in Figure 2. An image correctly classified by the network as pertaining to Class 1 was first divided into superpixels using a segmentation algorithm (in this case, the Simple Linear Iterative Clustering (SLIC) algorithm; see Section 2.3).

The segmented image was then used to generate a distribution of perturbed images, where superpixels were covered in black, at random. The perturbed image distribution was comprised of 10,000 images. Each of these was passed through the studied CNN model, generating the prediction probabilities for Class 0 and Class 1. This number of perturbed images was manually determined, by generating explanations repeatedly for a same given image until the resulting explanation weights were consistent. With 10,000 samples, the average difference between weights for multiple repeated explanations of the same image was typically on an order of magnitude between 0.0001 and 0.001, whereas the most relevant weights were typically in the range of 0.5–0.9. This indicates that 10,000 samples seem to be enough for reproducibility purposes in this case.

The perturbed images and probabilities were then presented to a linear regression model that estimated how each superpixel contributed to the overall prediction. In other words, the perturbed images formed a vicinity around the original image where the linear model (the regression) learned to approximate the non-linear model (the CNN).

Throughout, this manuscript the weights output by the linear regression are plotted with a blue-red color map, where blue/red pixels indicate positive/negative weights. Color intensity is proportional to the absolute value of the weight. As such blue regions indicate superpixels that contribute towards the correct classification, and red indicate the opposite. This LIME implementation allows the user to generate explanations for each class separately if requested.

### 2.3. Segmentation Algorithms

The choice of segmentation algorithms for superpixel generation can greatly influence the resulting explanations of the proposed XAI approach. The current implementation of LIME had three options for segmentation algorithms, all from the scikit-image Python library.

**Figure 3:**
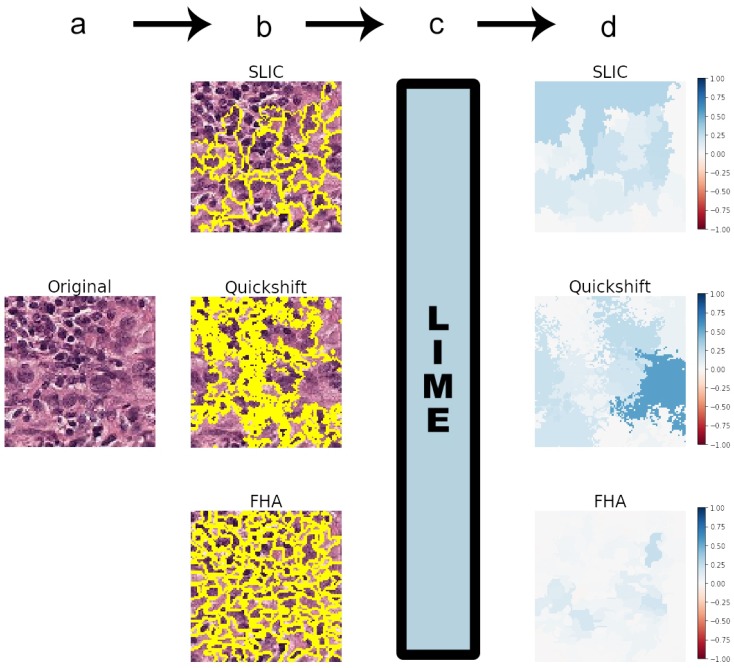
Methodology flowchart, showing the segmentation algorithms included in the LIME implementation: the Simple Linear Iterative Clustering (SLIC), quickshift, and Felzenszwalb (FHA) algorithms. Panel (**a**) shows the first step, where an image is taken to be classified by the chosen CNN model. In Step (**b**), this image is segmented into superpixels by any chosen segmentation algorithm. Step (**c**) comprises inputting the image and the defined superpixels into the LIME algorithm. Step (**d**) involves plotting the outputs of LIME as a heat map with a blue-red color map in the [−1, 1] range. This heat map was the generated explanation for the CNN classification of the image in Step (a). As seen in Panel (d), explanations were very dependent on the segmentation algorithms.

Figure 3 shows the general flowchart of the methodology employed in this study, including an example of a true positive (image of Class 1 correctly predicted). The example presented in Figure 3 used the standard parameters of the segmentation algorithms [14].

These algorithms are, namely:

1. Felzenszwalb’s [15] efficient graph-based image segmentation (FHA) generates an oversegmentation of an RGB image using tree-based clustering. The main parameter for indirectly determining the number of segments (superpixels) generated is “scale”, which sets an observation level.
2. Simple Linear Iterative Clustering (SLIC), by Achanta et al. [16], segments the image by using K-means clustering in the color space. There is a “number of segments” parameter that roughly tries to dictate the number of superpixels generated.
3. Quickshift, by Vedaldi et al. [17], performs segmentation by using a quickshift mode seeking algorithm to cluster pixels. There is no specific parameter for controlling the final number of superpixels.

Additionally, all three algorithms have a parameter “sigma” that defines the width of a Gaussian preprocessing step. Higher sigmas typically result in a smaller number of segments. This is the only parameter shared in common by all algorithms.

As the standard value of sigma for FHA in the library is 0.8, this was also used for the other two algorithms in most experiments.

To allow for a more systematic comparison of the explanations, only images where all algorithms generated the same number of superpixels were used for this study. The segmentation parameters had to be fine tuned for this purpose. As quickshift allowed the least control over the number of segments, it was always used as a baseline, and the other two algorithms were fine tuned to match the same amount of superpixels.

#### Squaregrid

The sensitivity of these algorithms to variations in texture and color and their own variables raises the question of how the best set of parameters can be determined. Although this is somewhat arbitrary, the idea is that the best set of parameters corresponds to the most interpretable explanations. Simply using the standard values for segmentation might not produce a meaningful explanation.

As an alternative approach, a new method was proposed to segment each studied image into grids of 9, 16, 36, 64, 144, 256, and 576 equally-sized squares. On the one hand each of those square segments did not hold the contextual meaning of a typical superpixel, but on the other hand, this guaranteed that every image was divided into exactly the same number of segments on the same positions.

These square segments were then passed to the LIME algorithm like the usual superpixels would be. The resulting weight heat maps gave a rough idea of what subregions of the image were most relevant for a given classification. The finer grids, with a larger number of small square segments, provided a more fine-grained view.

**Figure 4:**
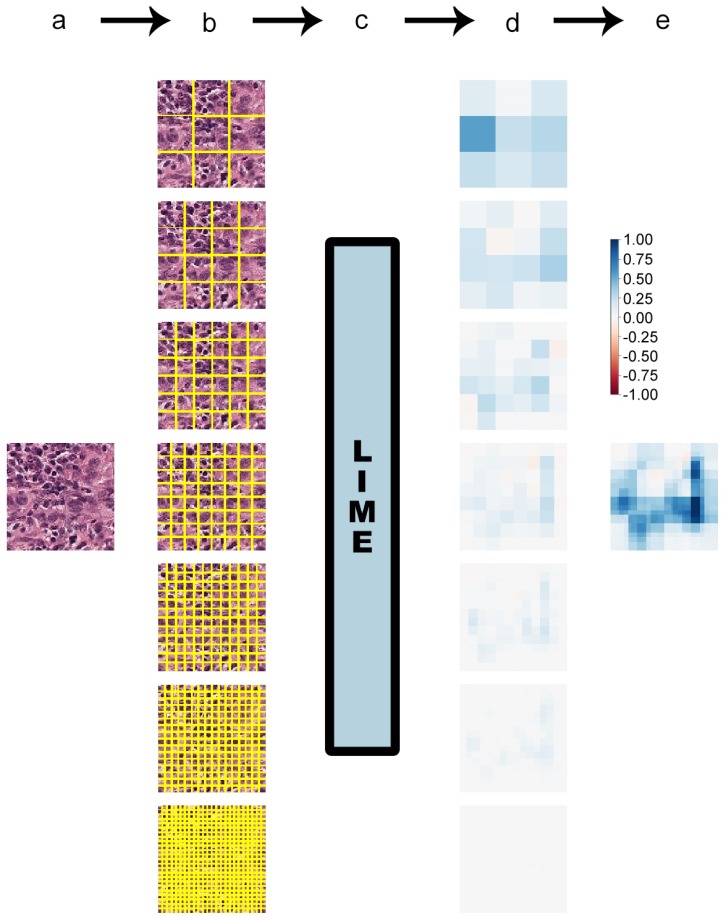
Diagram of the squaregrid method in four steps. The flowchart is identical to the previously-presented one, but instead of superpixels, the image was progressively divided into finer square grids. Seven grids were used, ranging from nine squares to 576. Explanation heat maps were generated for each of these grids, and a final heat map was computed as the sum of the seven previous heat maps.

The heat maps of all grids were then added, generating a final heat map. Although this procedure certainly should not be expected to generate explanations as detailed as the more advanced superpixel algorithms, the rough baseline they provide could be a useful approximation that is independent of parameter fine-tuning and easier to understand across images since the number of segments is guaranteed to always be the same. For the sake of brevity, this algorithm will be termed “squaregrid” throughout this manuscript. Figure 4 contains an example of this approach.

A relevant question is comparing the proposed squaregrid and the SLIC, quickshift and FHA algorithms with regards to which areas of an image they consider to be relevant in favor or against a classification. If all generate roughly similar explanations, squaregrid can become an interesting approach since it does not require parameter fine-tuning. This comparison is provided in Section 3.2 and Section 3.3.

### 2.4. Models

#### 2.4.1. Model1

The main goal of this work is to generate explanations for a CNN with good classification performance, rather than optimizing and training CNN architectures for the task from scratch. Any existing high-performing classifier could be used for this purpose. As such, the publicly-available models in the P-CAM-based competition [13] were assessed regarding their performance. The evaluation metric in this case was the Area Under the receiver operating characteristic Curve (AUC). Since the experiments run for this project were implemented in Keras [18], a publicly-available Keras CNN model with high AUC was selected [19]. This corresponds to the public submission by the user Marsh. More precisely, the h5 file with the trained CNN weights was downloaded, and the model was then reimplemented using these weights for the purpose of running the experiments. As this model is a custom architecture with seemingly no specific name, it will be termed “Model1” throughout this manuscript. This model consisted of a CNN with an AUC of 0.9528 on the public test set. It was a convolutional neural network with the following layers:

- Input layer (96×96×3 input dimensions)
- Convolution layer (32 filters, 3×3 kernels, ReLU activation)
- Convolution layer (32 filters, 3×3 kernels, ReLU activation)
- Convolution layer (32 filters, 3×3 kernels, ReLU activation)
- Max pooling (2×2 pooling)
- Dropout (30%)
- Convolution layer (64 filters, 3×3 kernels, ReLU activation)
- Convolution layer (64 filters, 3×3 kernels, ReLU activation)
- Convolution layer (64 filters, 3×3 kernels, ReLU activation)
- Max pooling (2×2 pooling)
- Convolution layer (128 filters, 3×3 kernels, ReLU activation)
- Convolution layer (128 filters, 3×3 kernels, ReLU activation)
- Convolution layer (128 filters, 3×3 kernels, ReLU activation)
- Max pooling (2×2 pooling)
- Flatten
- Dense (256 neurons, ReLU activation)
- Dropout (30%)
- Dense (2 neurons, softmax activation)

In total, there were 1,661,186 trainable weights.

#### 2.4.2. VGG19

In order to evaluate the proposed squaregrid performance with different classification models, another commonly-used and already established state-of-the-art image classification architecture was also used. The VGG19 architecture was selected for this task. This corresponds to the public submission by the user Maxime Lenormand [20]. Throughout the manuscript, this model will be referred to as “VGG19”, so as to differentiate it from Model1. This model consisted of a CNN with an AUC of 0.9683 on the public test set. It included the following layers:

- Input layer (96×96×3 input dimensions)
- Convolution layer (64 filters, 3×3 kernels, ReLU activation)
- Convolution layer (64 filters, 3×3 kernels, ReLU activation)
- Max pooling (2×2 pooling, stride = (2, 2))
- Convolution layer (128 filters, 3×3 kernels, ReLU activation)
- Convolution layer (128 filters, 3×3 kernels, ReLU activation)
- Max pooling (2×2 pooling, stride = (2, 2))
- Convolution layer (256 filters, 3×3 kernels, ReLU activation)
- Convolution layer (256 filters, 3×3 kernels, ReLU activation)
- Convolution layer (256 filters, 3×3 kernels, ReLU activation)
- Convolution layer (256 filters, 3×3 kernels, ReLU activation)
- Max pooling (2×2 pooling, stride = (2, 2))
- Convolution layer (512 filters, 3×3 kernels, ReLU activation)
- Convolution layer (512 filters, 3×3 kernels, ReLU activation)
- Convolution layer (512 filters, 3×3 kernels, ReLU activation)
- Convolution layer (512 filters, 3×3 kernels, ReLU activation)
- Max pooling (2×2 pooling, stride = (2, 2))
- Convolution layer (512 filters, 3×3 kernels, ReLU activation)
- Convolution layer (512 filters, 3×3 kernels, ReLU activation)
- Convolution layer (512 filters, 3×3 kernels, ReLU activation)
- Convolution layer (512 filters, 3×3 kernels, ReLU activation)
- Max pooling (2×2 pooling, stride = (2, 2))
- Dense (1 neurons, sigmoid activation)

In total, there were 20,024,897 trainable weights.

## 3. Results and Discussion

### 3.1. Generating Explanations

The P-CAM dataset has more than 200,000 images, and any of them could be analyzed from the perspective of generating explanations with the above-described procedure. However, some images in the dataset do share similar color and texture characteristics, and the overall behavior of the explanations was consistent for those.

**Figure 5:**
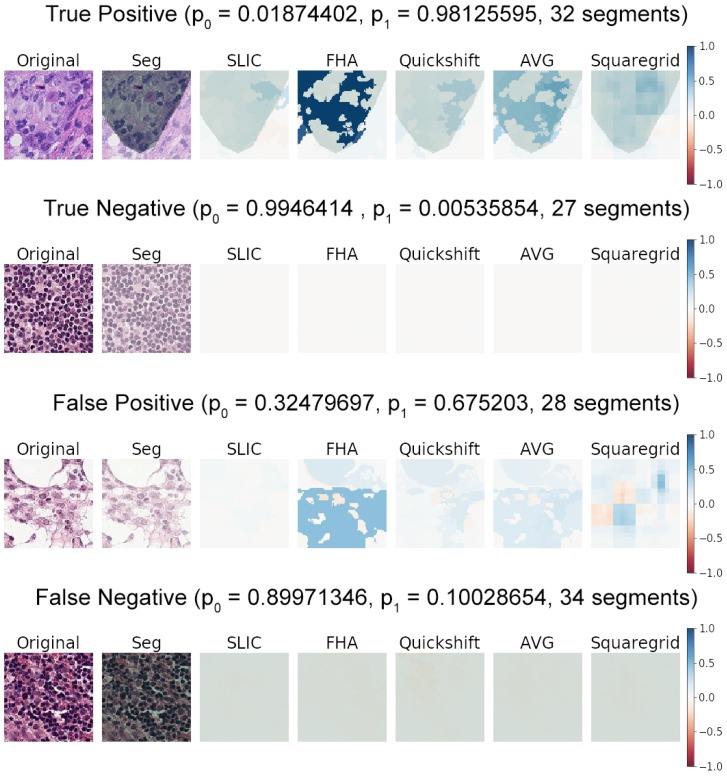
Examples of predictions by Model1, including a true positive, true negative, false positive, and false negative. The probabilities predicted for each class are also displayed as $p_{0}$ for Class 0 (no tumor tissue in the center 32 by 32 pixel square) and $p_{1}$ for Class 1 (tumor tissue present in the center). Explanations are plotted as weight heat maps for the respective predicted classes, with blue indicating positive weights (in favor of the prediction) and red indicating negative weights (against the prediction). Segmentation (Seg) (green transparent overlays) represent the medical annotation for a given image. AVG represents the arithmetic average of the SLIC, FHA, and quickshift heat maps.

The results showed the general behavior observed after generating and manually examining roughly forty sample images from the dataset. Figure 5 shows the basic characteristics observed for true positives, true negatives, false positives, and false negatives of classifications by Model1.

Although it is not possible to guarantee that the segmentation algorithms will divide the image on the same number of superpixels each time, they roughly seemed to divide the patches between 20 and 40 superpixels for similar ranges of parameters. Such ranges are generally in the vicinity of FHA scales between 200 and 600, an SLIC number of segments between 20 and 70, and a sigma of 0.8 for all algorithms. Fine-tuning these parameters by hand often converged to these values for the images sampled.

Attempting to generate a large number of superpixels (hundreds or thousands) quickly showed that each superpixel would corresponded to a very small region of the image, with few pixels, which holds no relevant visual information for the classification. As such, they understandably were associated with negligible explanation weights, and the heat maps were basically all white. The same was true for the finest square grids, as seen in Figure 4 for the grid with 576 squares (bottom). The heat map was practically all white. This largely indicated that it was not necessary to increase the computation times by generating even finer square grids (for instance, grids with 1024 or 2304 squares with this same approach) or manually adjust segmentation parameters to produce extremely large amounts of superpixels.

Positive classifications (where the class predicted was 1), whether true or false positives, generally presented explanations with large positive weights and virtually invisible negative weights if a symmetrical color scale was used. This happened in stark contrast with the true/false negatives, which presented an explanation with very small positive/negative weights if the same color scale was used. This is shown in Figure 5 by the nearly white heat maps of the true negative and false negative examples.

This behavior was very consistent across positive and negative classifications. It seemed to indicate that the CNN did not particularly identify any structures as strongly indicative of normal tissue; otherwise, large positive weights (strong blue areas) would be noticeable somewhere on the true/false negative heat maps.

It seems, rather, that the CNN had learned to identify structures that indicated the presence of tumors in the center of the image. This was indicated by the blue areas in the explanation heat maps. The absence of any such indicative structures in the explanation heat maps correlated to the prediction of Class 0. This followed human intuition to some degree, where a pathologist typically scans an image for indications of abnormal structures/tumor tissue, classifying it as healthy if nothing abnormal is found.

### 3.2. Comparing SLIC, FHA, and Quickshift

**Figure 6:**
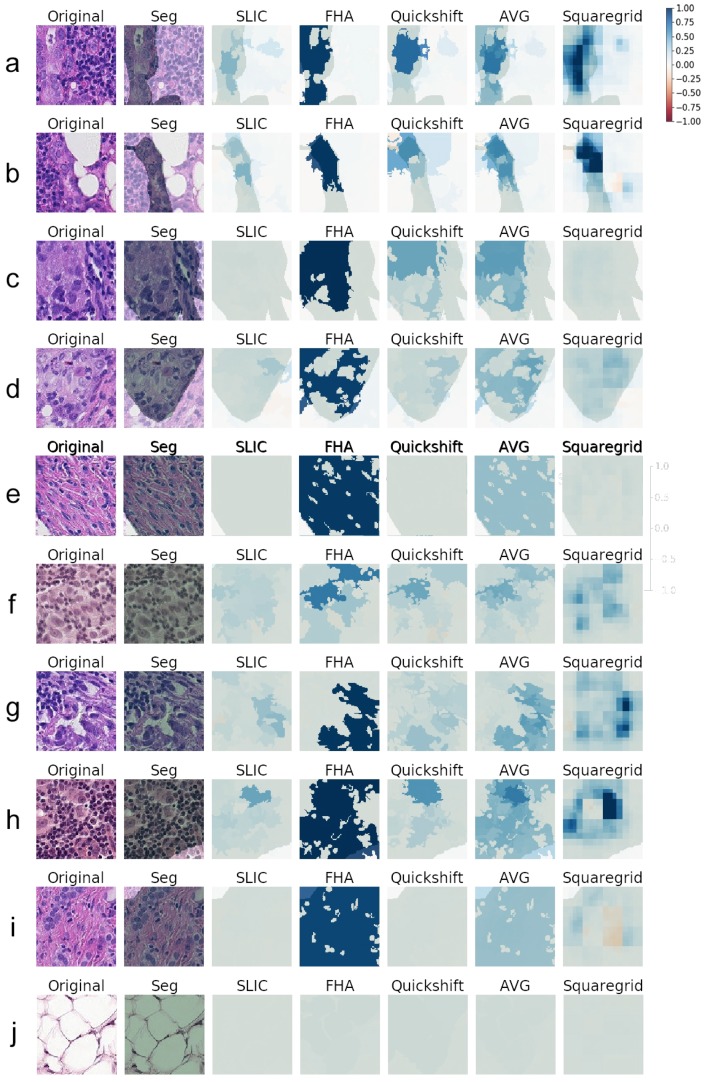
(**a**–**j**) Explanations generated for several images correctly predicted as Class 1 (true positives) by Model1. Medical Segmentation (Seg) in transparent green. All heat maps used the same red-blue color scale with the same limits of [−1,1]. AVG corresponds to the arithmetic average of the SLIC, FHA, and quickshift heat maps.

**Figure 7:**
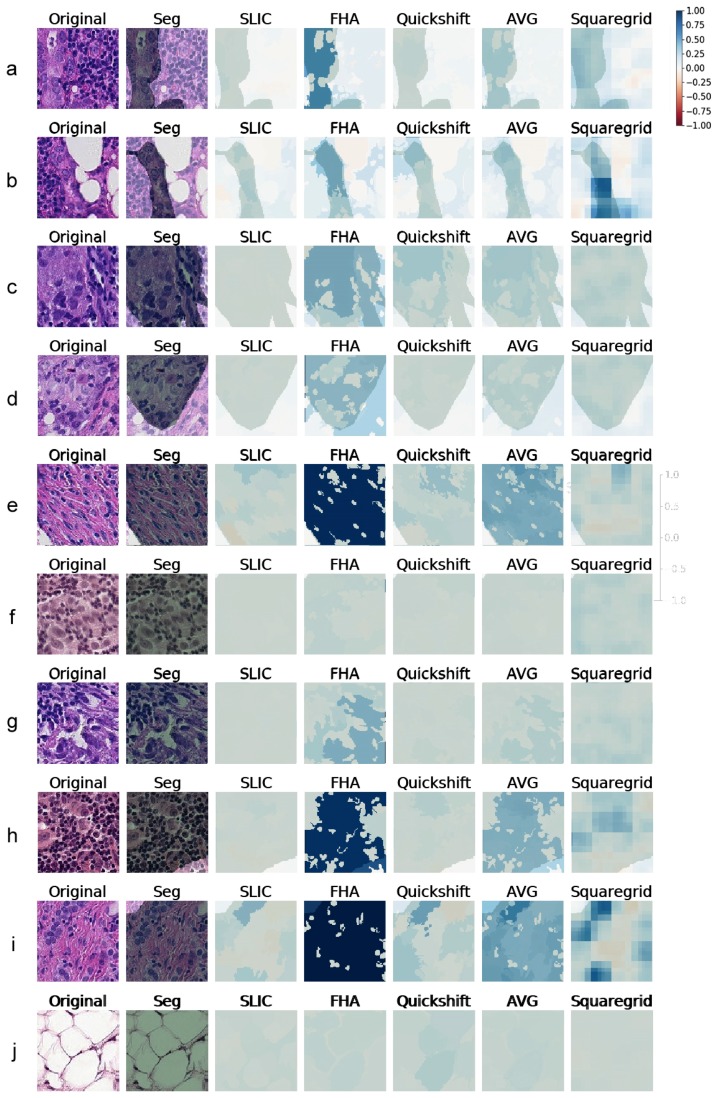
(**a**–**j**) Explanations generated for several images correctly predicted as Class 1 (true positives) by VGG19. Medical Segmentation (Seg) in transparent green. The color scheme is the same used in Figure 6.

Since the true positive predictions presented the explanations with the largest weights, indicating that the LIME was finding relevant superpixels, more true positive explanations were generated. Figure 6 contains 10 examples of explanations generated for Model1, with the respective heat maps for each segmentation algorithm. Similarly, Figure 7 shows explanations generated for VGG19 classifications. All weight heat maps are plotted with the same color scale used so far in the manuscript (symmetrical, red-blue, with limits from −1–1). It is worth stressing that the superpixels used to generate explanations both for VGG19 and Model1 were the same.

Although the images varied considerably in textures and colors, some key aspects became apparent. When the SLIC, quickshift, and FHA algorithms were forced to output the same number of segments, there were three main situations that seemed to arise for true positives:

- Cases where the medical annotation (corresponding to metastatic tissue) nearly or fully covered the patch and textures and colors were very homogeneous throughout the patch:

In these cases, no individual superpixel can explain the classification, as all regions of the patch were similar. This resulted in low weights all around, seen as white areas on the explanations. The one exception seemed to be the FHA algorithm that sometimes clumped all the regions similar in texture and color into one giant superpixel. Since this superpixel covered most of the metastatic tissue, it made sense that it ended up having a very high weight on the LIME explanation (often above 0.95).

Examples of this behavior can be seen in Figure 6 and Figure 7, Panel j. Panel e also shows a similar behavior, although explanations generated for VGG19 gave importance to some subregions that did not occur for the explanations of Model1.

- Cases where the medical annotation nearly or fully covered the patch, but there were multiple different textures and colors:

For these instances, the explanations often showed sub-regions of the patch that had higher weights and often corresponded to certain shapes, colors, or textures. Figure 6 (Panels f, g, h, i) and Figure 7 (Panels g, h, i), demonstrate this particular behavior. This seemed to indicate that whereas the visual information of the entire patch was relevant to pathologists, the neural networks were making classifications mostly influenced by specific smaller regions and characteristics rather than the entire patch.

- Cases where the medical annotation was a smaller subregion of the patch:

Figure 6 and Figure 7, Panels a–d, show examples of this case. Notably, the generated explanations highlighted higher weight areas always contained within the area corresponding to the segmentation, showing agreement between the LIME explanations and the pathologist annotations.

Like in the previously-discussed case, it seemed as if the network could generally make a classification/decision based on less visual information than the pathologist, as the methodology points to superpixels with high explanation weights, meaning that those small areas account for much of the CNN decisions, both for Model1 and VGG.

Overall, the segments found by the SLIC methodology seemed to result in the lowest relevance weights according to LIME, across both CNN models, as seen, for instance, in Panels b, e, f, g, h, and i of Figure 6.

SLIC, FHA, and quickshift seemed, however, to agree roughly on which areas of each image were important, in most cases. The darkest blue area of each heat map seemed to always have at least some overlap. By creating a new heat map consisting of the average of the three, the darkest blue areas denoting important structures became increasingly more detailed.

Row j of Figure 6 shows an image with a very different texture that occurred more rarely in the dataset, with several large white blobs. Although those were not uncommon in small numbers (such as the image in Row b), this image in particular was completely covered by them. Quickshift, FHA, and SLIC resulted in explanations with much lower weights for this case, despite the fact that the CNN model correctly classified this patch with a corresponding softmax output over 0.98. Cases were this happened seemed to be in the minority, however.

### 3.3. Squaregrid

The squaregrid approach, despite generating less detailed explanations, seemed to agree mostly with the other methods on the most relevant areas of the image, especially when compared to the averaged heat maps.

Each individual square of the different-sized grids did not hold much relevance on its own. This was expected since the squares did not particularly follow the textures and color changes of each image. Therefore, they did not hold any special context information, and there was no reason to imagine they would explain a given prediction well individually. However, by overlapping grids of different sizes, the positive and negative weights added up constructively/destructively, generating a profile that often matched the other heat maps to a remarkable degree. Rows b, e, f, h, and i of Figure 6 are particularly striking examples of this. It is notable that such a simple segmentation, devoid of parameters, generated interpretable results such as these through the LIME methodology.

The variation observed among quickshift, FHA, and SLIC was possibly caused by their sensitivity to segmentation parameters. The shape of the superpixels was highly variable and could influence the result greatly.

The squaregrid approach, on the other hand, did not have these issues. For example, Panels h and i of Figure 6 and Figure 7 show a case where the squaregrid explanation seemed to highlight enlarged nuclei with inconsistent shapes/colors/textures, whereas the other three algorithms did not point to the same regions with the same intensity (although the VGG19 model seemed to identify relevant regions more than Model1 in those cases).

### 3.4. Meaningfulness of Explanations

Comparing the generated explanations for both CNN models and the medical opinions showed that generally speaking, the explanations were often contained within the areas defined by the annotations. This can be seen throughout the examples of Figure 6 and Figure 7. As previously discussed, this indicated that both CNN models studied seemed to be generally focusing on areas that agreed with the opinions of medical experts/pathologists.

Given the slight degree of arbitrary fine-tuning that is possible with the parameters of the explanation algorithms (especially the segmentation methods), it is likely that having a parameter sweep, rather than fixed values, could aid in generating more meaningful explanations as well.

**Figure 8:**
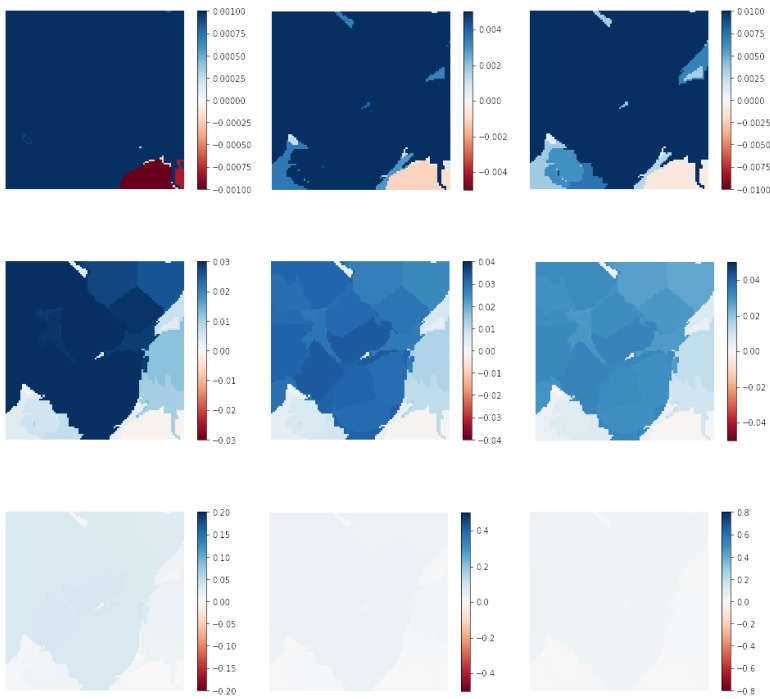
Heat map of Row j, Figure 6, replotted several times with color scale limits varying from [−0.001, 0.001]–[−0.8, 0.8].

As a simple example, Figure 8 shows various increasing limits for the color scale in the heat maps for Patch j in Figure 6. For previous examples, the color scale limits used were always [−1, 1]. However, for some images with very small weights, varying this range can aid in highlighting relevant regions. In Figure 8, the limits of the colormap were varied from [−0.001, 0.001]–[−0.8, 0.8]. A pathologist using this XAI methodology as a decision support tool could then apply this approach on every patch where deemed necessary.

### 3.5. Medicine/Biology Basis

One of the most important aspects of these results is that they stem from a very simple binary label, as described previously in the Materials and Methods (Section 2.1). The dataset labeling did not contain any detailed information of the tumor localization besides the presence of at least one pixel of tumor on the 32×32 pixel center of the patch. Remarkably, however, the CNN models learned how to identify relevant biological structures. Although it was already noted that the explanations, in general, overlapped with the medical annotations, the CNN models seemed to often focus on some specific characteristics of the tissues.

For example, one common structure on the patches was small dark purple blobs with very circular shapes, usually present in great numbers. They were visible in several patches already shown in Figure 1 and Figure 5, Figure 6 and Figure 7, but rarely seemed to be the main focus of LIME explanations. Areas containing such dark purple circles seemed to either appear as white or light blue in the averaged and squaregrid colormaps. This seems to suggest that this type of structure is generally not indicative of tumors. The true positive instance in Figure 5 and Rows a, b, e, f, and g of Figure 6 are good examples of this.

On the other hand, the areas with dark blues in the explanation often corresponded to changes in texture and color to lighter shades of pink/purple and often to very large, less consistently-shaped lighter purple blobs. This seemed to be especially true for squaregrid heat maps. The best examples of this are Rows b, f, g, h, and i of Figure 6, although the same behavior can be observed in most other patches as well. The presence of those deformed enlarged purple blobs seemed to indicate the presence of tumor tissue for the CNN model.

In some images, these enlarged nuclei overlapped, and this seemed to factor in the weight given by explanations, especially for squaregrid (Rows b and h of Figure 6).

This is similar to the example shown in the Camelyon16 challenge website [21], where the tissue marked as normal had many of the small dark purple circles and the metastatic tissue had more enlarged pink textures and large lighter purple nuclei that were less consistently shaped.

Furthermore, the Elston–Ellis scale used for histological grading and differentiation of breast cancers [22] focuses on some semiquantitative features that go along similar lines to this discussion. For instance, nuclear pleomorphism, the degree to which nuclei are uniform in size or varying considerably, is one of the features considered, with a marked variation receiving the highest score. This definitely seemed to be one of the aspects relevant in the explanations generated in this manuscript.

Mitotic count is another feature considered in the Elston–Ellis scale. Whether the enlarged overlapping nuclei cited two paragraphs above were indicative of mitotic activity can be confirmed by pathologists, it might explain why they seemed to be considered relevant in the LIME explanations. Indeed, future projects should aim to compare this type of study to the opinion of medical experts, as that is the gold standard for such tasks.

## 4. Conclusions

In this manuscript, the LIME methodology was used to generate explanations for two CNN models classifying histology WSI patches for the presence or absence of metastases, which appears to be among the first applications of this XAI technique directly on a medical image classification problem. It is important to note that the same approach can be used for any type of AI model, since LIME is a model-agnostic methodology.

Different segmentation algorithms were used for superpixel generation, including a new proposed simple squaregrid approach. After comparing the behavior of each segmentation algorithm, explanations were generated for a series of true positive predictions. The characteristics of these images were then analyzed, and some patterns on which structures influence positive predictions were noted. The methodology described and used in this work can be adapted to other medical imaging classification tasks. The explanations were also compared to medical annotations on the same images, generally finding both in agreement.

The main goal of this work was to propose a methodology to generate explanations for classification of medical image patches, without necessarily expecting the generated explanations to mimic human expert knowledge. However, the analysis seems to initially demonstrate that the AI model in this case was following at least some basic premises of the human approach for the same task; that is, focusing on abnormal tissue when predicting the presence of tumors, with the abnormalities often consisting of enlarged and deformed nuclei or cell bodies within the tissue. Furthermore, the explanations in general highlighted areas inside the regions defined by the medical annotations.

The analysis presented greatly motivated collaborations with expert pathologists in future studies to evaluate the AI explanations more in depth and with a larger ensemble of models, as well as applying the approach here presented to other medical imaging datasets.
